# Supplementary material for: Changes of Thermostability, Organic Solvent, and pH Stability in Geobacillus zalihae HT1 and Its Mutant by Calcium Ion
Source: Int J Mol Sci. 2019 May 24;20(10):2561. doi: 10.3390/ijms20102561 (PMC6566366; doi:10.3390/ijms20102561)
Supplement: Supplementary file 1 [file ijms-20-02561-s001.pdf]

```
CLUSTAL O(1.2.4) multiple sequence alignment


wt_HT1_unmutated_sequence      ------------------------------------------------------------	0
5M_Full_Sequence               GGTCTGCTAGCTGTGGTATGGCTGTGCAGGTCGTAATCACTGCATAATTCGTGTCGCTCA	60
                                                                                           

wt_HT1_unmutated_sequence      ------------------------------------------------------------	0
5M_Full_Sequence               AGGCGCACTCCCGTTCTGGATAATGTTTTTTGCGCCGACATCATAACGGTTCTGGCAAAT	120
                                                                                           

wt_HT1_unmutated_sequence      ------------------------------------------------------------	0
5M_Full_Sequence               ATTCTGAAATGAGCTGTTGACAATTAATCATCGGCTCGTATAATGTGTGGAATTGTGAGC	180
                                                                                           

wt_HT1_unmutated_sequence      ------------------------------------------------------------	0
5M_Full_Sequence               GGATAACAATTTCACACAGGAAACAGTATTCATGTCCCCTATATCTAGACATCATCATCA	240
                                                                                           

wt_HT1_unmutated_sequence      -------------------------GCATCCCTACGCGCCAATGATGCACCGATTGTGCT	35
5M_Full_Sequence               TCATCATCTGGTTCCGCGTGGATCCGCATCCCTACGCGCCAATGATGCACCGATTGTGCT	300
                                                        ***********************************

wt_HT1_unmutated_sequence      TCTCCATGGGTTTACCGGATGGGGACGAGAGGAAATGTTTGGATTCAAGTATTGGGGCGG	95
5M_Full_Sequence               TCTCCATGGGTTTACCGGATGGGGACGAGAGGAAATGTTTGGATTCAAGTATTGGGGCGG	360
                               ************************************************************

wt_HT1_unmutated_sequence      CGTGCGCGGCGATATCGAACAATGGCTGAACGACAACGGTTATCGAACGTATACGCTGGC	155
5M_Full_Sequence               CGTGCGCGGCGATATCGAACAATGGCTGAACGAGAACGGTTATCGAACGTATACGCTGGC	420
                               ********************************* **************************

wt_HT1_unmutated_sequence      GGTCGGACCGCTCTCGAGCAACTGGGACCGGGCGTGTGAAGCGTATGCTCAGCTTGTCGG	215
5M_Full_Sequence               GGTCGGACCGCTCTCGAGCAACTGGGACCGGGCGTGTGAAGCGTATGCTCAGCTTGTCGG	480
                               ************************************************************

wt_HT1_unmutated_sequence      CGGGACGGTCGATTATGGGGCAGCCCATGCGGCAAAGCACGGCCATGCGCGGTTTGGCCG	275
5M_Full_Sequence               CGGGACGGTCGATTATGGGGCAGCCCATGCGGCAAAGCACGGCCATGCGCGGTTTGGCCG	540
                               ************************************************************

wt_HT1_unmutated_sequence      CACTTATCCCGGCCTGTTGCCGGAATTGAAAAGGGGTGGCCGCATCCATATCATCGCCCA	335
5M_Full_Sequence               CACTTATCCCGGCCTGTTGCCGGAATTGAAAAGGGGTGGCCGCATCCATATCATCGCCCA	600
                               ************************************************************

wt_HT1_unmutated_sequence      CAGCCAAGGGGGGCAGACGCCCCGCATGCTTGTCTCGCTCCTAGAGAACGGAAGCCAAGA	395
5M_Full_Sequence               CAGCCAAGGGGGGCAGAACCCCCGCATGCTTGTCTCGCTCCTAGAGAACGGAAGCCAAGA	660
                               *****************  *****************************************

wt_HT1_unmutated_sequence      AGAGCGGGAGTACGCCAAGGCGCATAACGTGTCGTTGTCACCGTTGTTTGAAGGTGGACA	455
5M_Full_Sequence               AGAGCGGGAGTACGCCAAGGCGCATAACGTGTCGTTGTCACCGTTGTTTGAAGGTGGACA	720
                               ************************************************************

wt_HT1_unmutated_sequence      TCATTTTGTGTTGAGTGTGACGACCATCGCCACTCCTCATGACGGGACGACGCTTGTCAA	515
5M_Full_Sequence               TCATTTTGTGTTGAGTGTGACGACCATCGCCACTCCTCATGACGGGACGACGCTTGTCAA	780
                               ************************************************************

wt_HT1_unmutated_sequence      CATGGTTGATTTCACCGATCGCTTTTTTGACTTGCAAAAAGCGGTGTTGGAAGCGGCGGC	575
5M_Full_Sequence               CATGGTTGATTTCACCGATCGCTTTTTTGACTTGCAAAAAGCGGTGTTGGAAGCGGCGGC	840
                               ************************************************************

wt_HT1_unmutated_sequence      TGTCGCCAGCAACGTGCCGTACACGAGTCAAGTATACGATTTTAAGCTCGACCAATGGGG	635
5M_Full_Sequence               TGTCGCCAGCAACGTGCCGTACACGAGTCAAGTATACGATTTTAAGCTCGACCAATGGGG	900
                               ************************************************************
```

```
wt_HT1_unmutated_sequence      ACTGCGCCGCCAGCCGGGTGAATCGTTCGACCATTATTTTGAAGGGCTCAAGCGCTCCCC     695
5M_Full_Sequence               ACTGCGCCGCCAGCCGGGTGAATCGTTCGACCATTATTTTGATGGGCTCAAGCGCTCCCC     960
                               ****************************************** *****************

wt_HT1_unmutated_sequence      TGTTTGGACGTCCACAGATACCGCCCGCTACGATTTATCCGTTTCCGGAGCTGAGAAGTT     755
5M_Full_Sequence               TGTTTGGACGTCCACAGATACCGCCCGCTACGATTTATCCGTTTCCGGAGCTTTGAAGTT     1020
                               ****************************************************  * ****

wt_HT1_unmutated_sequence      GAATCAATGGGTGCAAGCAAGCCCGAATACGTATTATTTGAGTTTCTCTACAGAACGGAC     815
5M_Full_Sequence               GAATCAATGGGTGCAAGCAAGCCCGAATACGTATTATTTGAGTTTCTCTACAGAACGGAC     1080
                               ************************************************************

wt_HT1_unmutated_sequence      GTATCGCGGAGCGCTCACAGGCAACCATTATCCCGAACTCGGAATGAATGCATTCAGCGC     875
5M_Full_Sequence               GTATCGCGGAGCGCTCACAGGCAACCATTATCCCGAACTCGGAATGAATGCATTCAGCGC     1140
                               ************************************************************

wt_HT1_unmutated_sequence      GGTCGTATGCGCTCCGTTTCTCGGTTCGTACCGGAATCCGACGCTCGGCATTGACGACCG     935
5M_Full_Sequence               GGTCGTATGCGCTCCGTTTCTCGGTTCGTACCGGGAGCCGACGCTCGGCATTGACGACCG     1200
                               *********************************  *  **********************

wt_HT1_unmutated_sequence      ATGGTTGGAGAACGATGGCATTGTCAATACGGTTTCCATGAACGGTCCAAAGCGTGGATC     995
5M_Full_Sequence               ATGGTTGGAGAACGATGGCATTGTCAATACGGTTTCCATGAACGGTCCAAAGCGTGGATC     1260
                               ************************************************************

wt_HT1_unmutated_sequence      AAGCGATCGGATCGTGCCGTATGACGGGACGTTGAAAAAAGGGGTTTGGAATGATATGGG     1055
5M_Full_Sequence               AAGCGATCGGATCGTGCCGTATGACGGGACGTTGAAAAAAGGGGTTTGGAATGATATGGG     1320
                               ************************************************************

wt_HT1_unmutated_sequence      AACGTACAACGTCGACCATTTGGAAATCATCGGCGTTGACCCGAATCCGTCATTTGATAT     1115
5M_Full_Sequence               AACGTACAACGTCGACCATTTGGAAATCATCGGCGTTGACCCGAATCCGTCATTTGATAT     1380
                               ************************************************************

wt_HT1_unmutated_sequence      TCGCGCCTTTTATTTGCGGCTTGCCGAGCAGTTGGCGAGCTTGCAGCCTTAA--------     1167
5M_Full_Sequence               TCGCGCCTTTTATTTGCGGCTTGCCGAGCAGTTGGCGAGCTTGCAGCCTTAAGAATTCCC     1440
                               ****************************************************

wt_HT1_unmutated_sequence      ------------------------------------------------------------     1167
5M_Full_Sequence               GGGTCGACTCGAGCGGCCGCATCGTGACTGACTGACGATCTGCCTCGCGCGTTACGTATA     1500

wt_HT1_unmutated_sequence      -       1167
5M_Full_Sequence               G       1501
```

Figure S1. DNA sequence alignments of 5M mutated lipase and original sequence of T1 lipase for verification of mutation. The multiple sequences alignment was generated from https://www.ebi.ac.uk/Tools/services/rest/clustalo/result/clustalo-I20190408-165909-0083-17877188-p2m/aln-clustal_num. The mutated sequences are marked in red boxes.

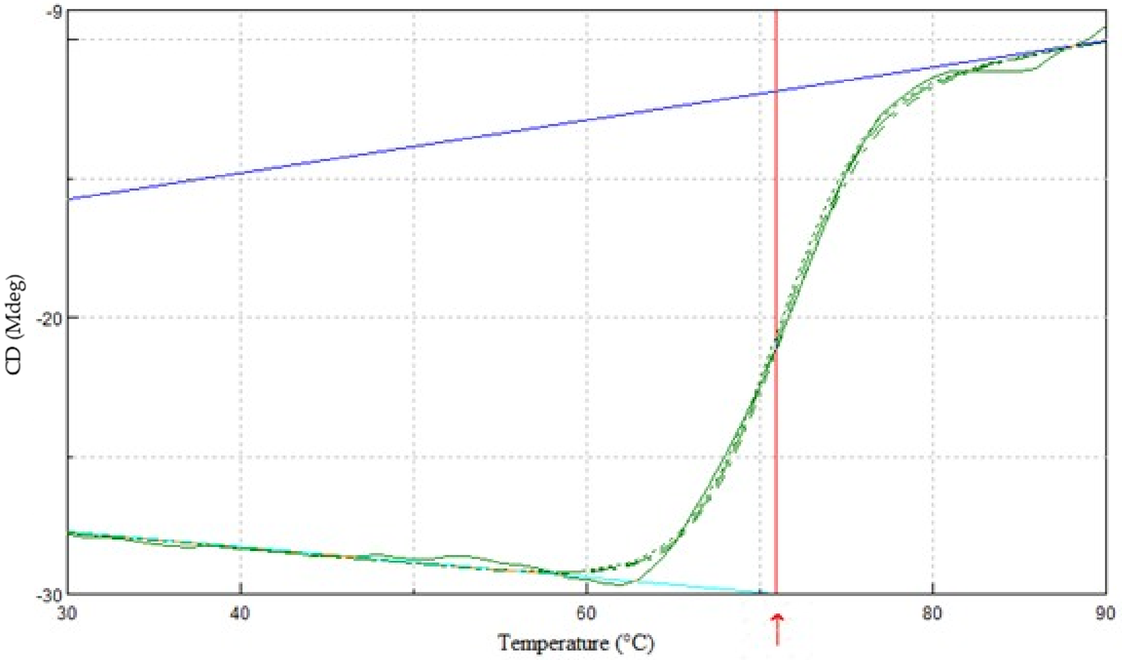

(a)

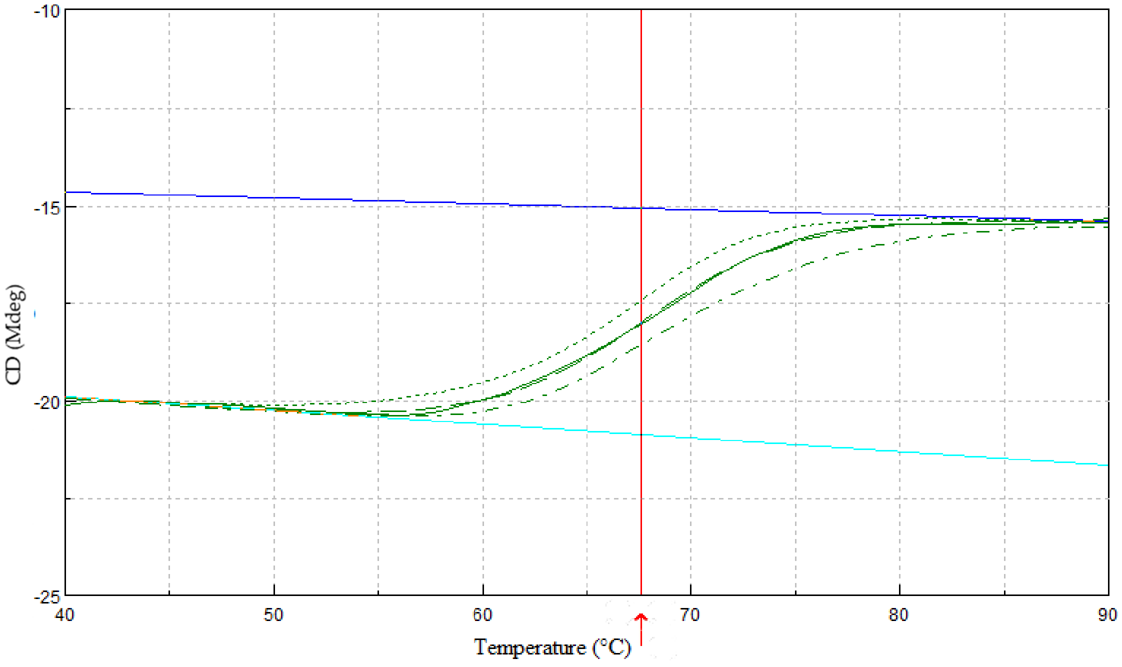

(b)

Figure S2. Melting temperature (T_m) curve of wt-HT1 and 5M mutant lipases using Circular Dichroism without calcium ion. (a) Temperature profile of wt-HT1 lipase. (b) Temperature profile of 5M mutant lipase. Red arrow indicated the thermal denaturation point of lipases.

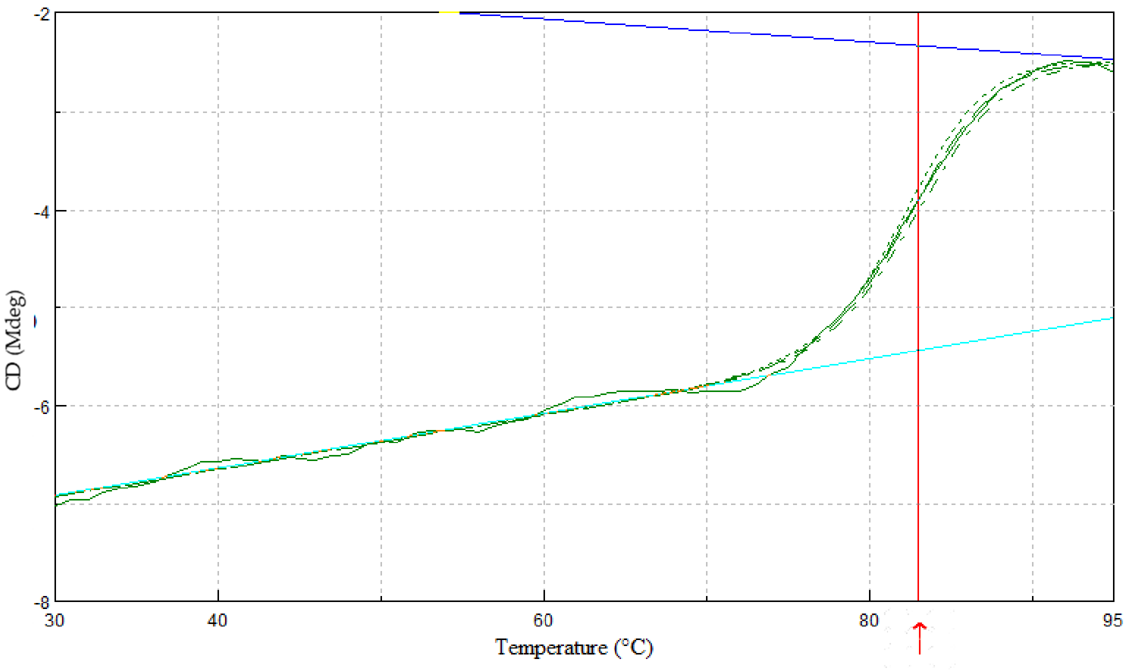

(a)

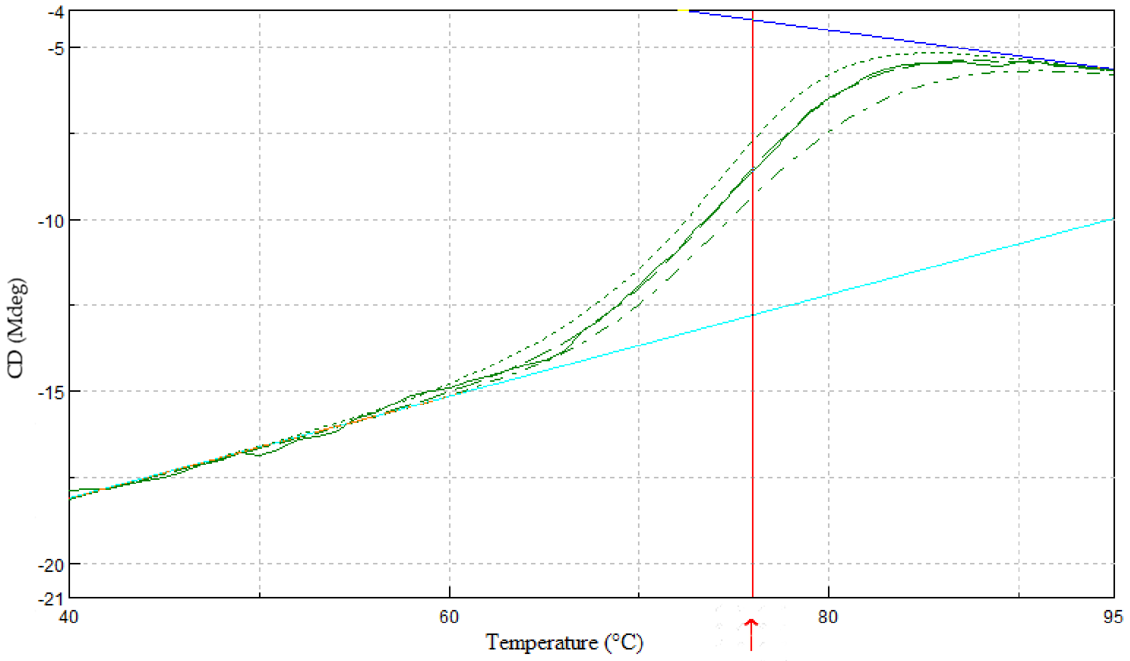

(b)

Figure S3. Melting temperature (T_m) curve of wt-HT1 and 5M mutant lipases using Circular Dichroism with the presence of 1 mM calcium ion. (a) Temperature profile of wt-HT1 lipase with 1 mM calcium ion. (b) Temperature profile of 5M mutant lipase with 1 mM calcium ion. Red arrow indicated the thermal denaturation point of lipases.

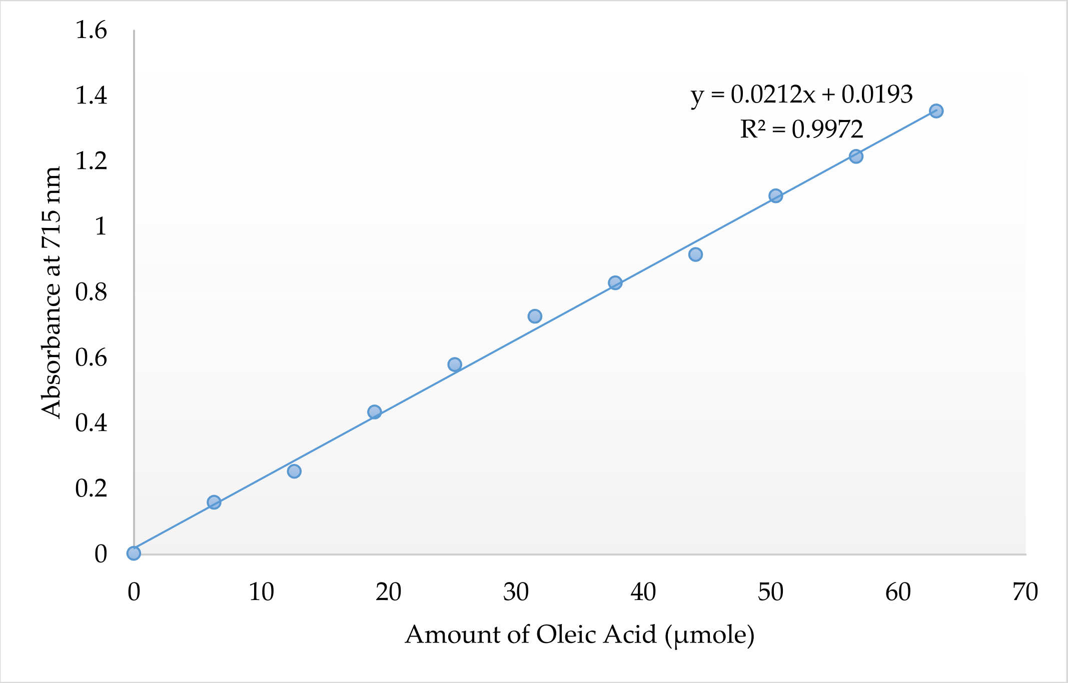

Figure S4. Oleic acid standard curve based on the method of Kwon and Rhee [59].
